# Association of systemic adverse reaction patterns with long-term dynamics of humoral and cellular immunity after coronavirus disease 2019 third vaccination

**DOI:** 10.1038/s41598-023-36429-1

**Published:** 2023-06-07

**Authors:** Makoto Yoshida, Yurie Kobashi, Takeshi Kawamura, Yuzo Shimazu, Yoshitaka Nishikawa, Fumiya Omata, Hiroaki Saito, Chika Yamamoto, Tianchen Zhao, Morihiro Takita, Naomi Ito, Kenji Tatsuno, Yudai Kaneko, Aya Nakayama, Tatsuhiko Kodama, Masatoshi Wakui, Kenzo Takahashi, Masaharu Tsubokura

**Affiliations:** 1 Faculty of Medicine, Teikyo University School of Medicine, Itabashi-ku, Tokyo, 173-8605 Japan; 2 Department of Radiation Health Management, Fukushima Medical University School of Medicine, Fukushima, Fukushima 960-1247 Japan; 3 Department of Internal Medicine, Serireikai Group Hirata Central Hospital, Ishikawa Country, Fukushima, 963-8202 Japan; 4 Isotope Science Centre, The University of Tokyo, Bunkyo-ku, Tokyo, 113-0032 Japan; 5 Laboratory for Systems Biology and Medicine, Research Centre for Advanced Science and Technology (RCAST), The University of Tokyo, Meguro-ku, Tokyo, 153-8904 Japan; 6 Genome Science & Medicine Laboratory, Research Center for Advanced Science and Technology (RCAST), The University of Tokyo, Meguro-ku, Tokyo, 153-8904 Japan; 7 Medical & Biological Laboratories Co., Ltd, Minato-ku, Tokyo, 105-0012 Japan; 8 Department of Laboratory Medicine, Keio University School of Medicine, Shinjuku-ku, Tokyo, 160-8582 Japan; 9 Teikyo University Graduate School of Public Health, Itabashi-ku, Tokyo, 173-8605 Japan; 10 Department of Pediatrics, Jyoban Hospital, Iwaki, Fukushima 972-8322 Japan; 11 Minamisoma Municipal General Hospital, Minamisoma, Fukushima 975-0033 Japan

**Keywords:** Immunology, Infectious diseases, Vaccines, Health services, Public health

## Abstract

The objective of this study was to clarify the impact of adverse reactions on immune dynamics. We investigated the pattern of systemic adverse reactions after the second and third coronavirus disease 2019 (COVID-19) vaccinations and their relationship with immunoglobulin G against severe acute respiratory syndrome coronavirus 2 spike 1 protein titers, neutralizing antibody levels, peak cellular responses, and the rate of decrease after the third vaccination in a large-scale community-based cohort in Japan. Participants who received a third vaccination with BNT162b2 (Pfizer/BioNTech) or mRNA-1273 (Moderna), had two blood samples, had not had COVID-19, and had information on adverse reactions after the second and third vaccinations (n = 2198) were enrolled. We collected data on sex, age, adverse reactions, comorbidities, and daily medicine using a questionnaire survey. Participants with many systemic adverse reactions after the second and third vaccinations had significantly higher humoral and cellular immunity in the peak phase. Participants with multiple systemic adverse reactions after the third vaccination had small changes in the geometric values of humoral immunity and had the largest geometric mean of cellar immunity in the decay phase. Systemic adverse reactions after the third vaccination helped achieve high peak values and maintain humoral and cellular immunity. This information may help promote uptake of a third vaccination, even among those who hesitate due to adverse reactions.

## Introduction

Coronavirus disease 2019 (COVID-19), caused by severe acute respiratory syndrome coronavirus 2 (SARS-CoV-2), which was first confirmed in China in December 2019, has resulted in 672 million infections and 6.7 million deaths as of January 19, 2023^1^. Vaccination is a key strategy for combating the COVID-19 pandemic. Messenger RNA (mRNA) vaccines mainly induce humoral and cellular immunity against COVID-19, preventing infection and severe disease^2^. Since July 2021, the third vaccination has been recommended due to waning vaccine effectiveness^3–6^. However, there are numerous challenges to address, including inequality in vaccine distribution^7^, waning effectiveness^8^, and adverse reactions after COVID-19 vaccination^9^. In addition, concerns about adverse reactions, including fever and fatigue, were essential factors in hesitation to receive the third vaccination^10–12^. Therefore, considering that the third vaccination is in progress worldwide, evaluating the short- and long-term effects of adverse reactions to COVID-19 vaccination is a crucial public health issue.

Several studies have been conducted on adverse reactions after COVID-19 vaccination. Adverse reactions can be influenced by age, sex, autoimmune disease, frequency of vaccine administration, injection technique, and use of immunosuppressants and non-steroidal anti-inflammatory drugs (NSAIDs)^9,13^. Notably, it has been suggested that by-products of type I interferons, which guide and promote the adaptive immune response of T and B cells, can cause adverse reactions^14–16^. Furthermore, some studies have reported that systemic adverse reactions correlate with humoral immunity^17–21^. However, others insist that humoral immunity does not correlate with adverse reactions^22–24^, and few studies have investigated the long-term effects of adverse reactions^17,25^. To the best of our knowledge, no studies have investigated the relationship between systemic adverse reactions after third vaccinations and the long-term dynamics of cellular immunity.

In Japan, more than 31 million infected patients and more than 63 thousand deaths have been reported as of January 19, 2023^26^. In Japan, a third vaccination was provided in December 2021 to healthcare workers and residents of geriatric facilities. Adults aged 65 years or older and more than 85 million people (67.9%) had received a third vaccination by January 19, 2023^27^. In Fukushima Prefecture, the Fukushima Vaccination Community Survey (FVCS), which is a series of antibody tests against COVID-19, has been conducted since the early phase of the pandemic^28^. The FVCS conducts antibody testing every 3 months, surveys adverse reactions after COVID-19 vaccination with approximately 2500 people under multi-sectional collaboration between local governments, universities, and hospitals, and reports the findings to the local population^11,20,29–36^. Therefore, this area is suitable for evaluating adverse reactions and immune dynamics, after third vaccinations, at the community level.

Although the third vaccination is in progress worldwide, information on the relationship between adverse reactions and humoral and cellular immunity after the third vaccination was limited. Therefore, this study aimed to clarify the impact of adverse reactions, particularly systemic adverse reactions, on immune dynamics. We investigated the pattern of systemic adverse reactions after the second and third vaccinations and their relationship with immunoglobulin G against spike 1 protein (IgG(S)) titers, neutralizing antibody (Nab) levels, peak cellular responses, and the rate of decrease after the third vaccination.

## Results

### Characteristics of participants and immunogenicity

Among the 2198 participants, the mean age, height, and weight were 53.5 ± 18.3 years, 161.4 ± 9.7 cm, and 61.8 ± 13.7 kg, respectively; and 1267 (57.6%) participants were female (Table 1). After the third vaccination, the median IgG(S) was 2221.4 AU/mL (interquartile range (IQR) 1318.8–3831.1) in the peak phase (T1: the median of the day from the third vaccination was 54 days) and decreased to 903.7 AU/mL (IQR 499.6–1685.4) in the decay phase (T2: the median of the day from the third vaccination was 145 days). The median Nab was 800 AU/mL (IQR 800.0–800.0) at T1 and decreased to 754.6 AU/mL (IQR 537.1–800.0) at T2. The median ELISpot was 11 (IQR 5–25) at T1 and 11 (IQR 5–25) at T2.

**Table 1:** Basic characteristics of participants (N = 2198).

	n (%)
Age, mean [SD]	53.5 [18.3]
Female	1267 (57.6)
Height, mean [SD]	161.4 [9.7]
Weight, mean [SD]	61.8 [13.7]
BMI	
Thin	106 (4.8)
Normal	1175 (53.5)
Overweight	586 (26.6)
Alcohol	963 (43.8)
Smoking	411 (18.7)
Daily medications	
Steroids	45 (2.1)
NSAIDs	163 (7.5)
Acetaminophen	52 (2.4)
Antihistamines	135 (6.2)
Immunosuppression	22 (1.0)
Biologics	11 (0.5)
Anticancer drugs	10 (0.5)
Comorbidities	
Hypertension	607 (27.6)
Diabetes	165 (7.5)
Asthma	106 (4.8)
Anaphylactic shock	18 (0.8)
Gout	64 (2.9)
Dyslipidemia	251 (11.4)
Rheumatoid arthritis	35 (1.6)
Respiratory disease	45 (2.0)
Cardiovascular disease	169 (7.7)
Second vaccination type (Moderna)	1 (0.1)
Adverse reactions after second vaccination	
Local pain	1248 (56.8)
Fever	610 (27.8)
Fatigue	1091 (49.6)
Headache	583 (26.5)
Muscle/joint pain	677 (30.8)
Diarrhea	49 (2.2)
Nausea	85 (3.9)
Dizziness	94 (4.3)
Third vaccination type (Moderna)	730 (33.5)
Adverse reactions after third vaccination	
Local pain	1358 (61.8)
Fever	653 (29.7)
Fatigue	1021 (46.4)
Headache	634 (28.8)
Muscle/Joint pan	698 (31.7)
Diarrhea	54 (2.5)
Nausea	92 (4.2)
Dizziness	93 (4.2)
After second vaccination	
T1	
IgG(S), median [IQR]	315.4 [172.1–546.2]
Nab, median [IQR]	91.0 [42.6–219.9]
T2	
IgG(S), median [IQR]	136.6 [80.4–225.4]
Nab, median [IQR]	38.5 [21.3–82.0]
After third vaccination	
T1	
IgG(S), median [IQR]	2221.4 [1318.8–3831.1]
Nab, median [IQR]	800.0 [800.0–800.0]
T spot (S), median [IQR]	11 [5–25]
T2	
IgG(S), median [IQR]	903.7 [499.6–1685.4]
Nab, median [IQR]	745.6 [537.1–800.0]
T spot (S), median [IQR]	11 [5–25]

### Groups according to adverse reactions

**Figure 1:**
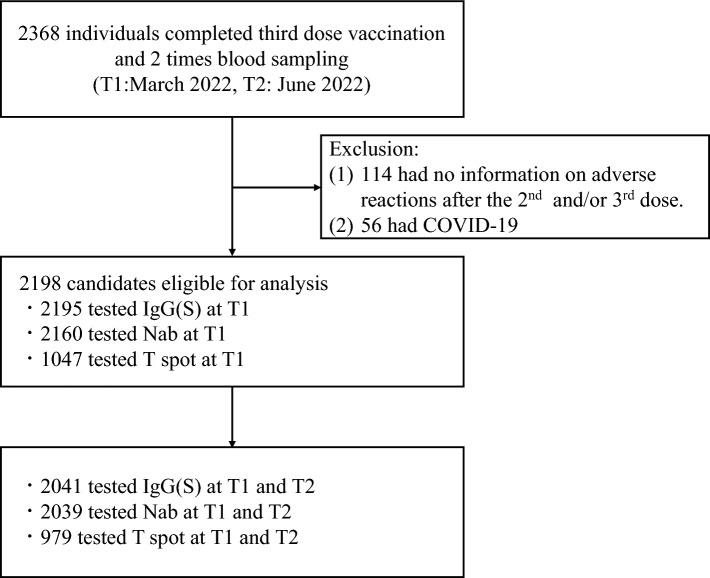
Eligible participants. Participants were included if they (i) completed the third COVID-19 mRNA vaccination (BNT162b2 or mRNA-1273) and (ii) completed blood sampling during the peak (T1: the median of the day from the third vaccination was 54 days) and decay phases (T2: the median of the day from the third vaccination was 145 days). We excluded participants without a record of adverse reactions after the second or third vaccination and those who had COVID-19 by T2. *COVID-19* coronavirus disease 2019.

After the second and third vaccinations, the rates of local reactions (56.8% and 61.8% after the second and third vaccinations, respectively) and systemic reactions (64.6% and 63.5% after the second and third vaccinations, respectively) were similar. The most reported systemic adverse reactions were fatigue (49.6% and 46.4% after the second and third vaccinations, respectively), muscle/joint pain (30.8% and 31.7% after the second and third vaccinations, respectively), fever (27.8% and 29.7% after the second and third vaccinations, respectively), and headache (26.5% and 28.8% after the second and third vaccinations, respectively). Of the 776 (35.3%) patients without systemic symptoms after the second vaccination, 539 (24.5%, Group 1) had no systemic symptoms after the third vaccination, 138 (6.3%, Group 3) had one, and 99 (6.3%, Group 6) had two or more symptoms (Fig. 1). Of the 511 (23.2%) patients with one systemic symptom after the second vaccination, 163 (7.4%, Group 2) had no systemic symptoms after the third vaccination, 150 (6.8%, Group 5) had one, and 198 (9.0%, Group 8) had two or more. Of the 911 (41.4%) patients with two or more systemic symptoms after the second vaccination, 101 (4.6%, group 4) had no systemic symptoms after the third vaccination, 177 (8.1%, group 7) had one, and 633 (28.8%, group 9) had two or more.

### Factors associated with patterns of systemic adverse reactions

The results of multinomial logistics regression based on Group 1 revealed that Group 1 participants were significantly older than all groups (Table 2). Group 1 was significantly associated with males compared to the groups with an exceptionally high number of systemic reactions (Groups 7–9), the groups with one or multiple systemic reactions after the second vaccination only (Groups 2 and 4), and the group with one systemic reaction at each vaccination (Group 5). Furthermore, receiving mRNA-1273 (Moderna) as the third vaccination was significantly lower in Groups 2 and 4 and higher in Group 3. Smoking habits (Groups 4, 7, and 9), alcohol consumption (Group 7), asthma (Groups 5, 7, and 9), use of anticancer drugs (Group 4), and body mass index (BMI) (Group 8) were significantly different from those in Group 1.

**Table 2:** Multinomial logistics regression analysis of differences among the groups.

	Group 1 (n = 539)	Group 2 (n = 163)		Group 3 (n = 138)		Group 4 (n = 101)		Group 5 (n = 150)		Group 6 (n = 99)		Group 7 (n = 177)		Group 8 (n = 198)		Group 9 (n = 633)	
	Base	B (95% CI)	p-value	B (95% CI)	p-value	B (95% CI)	p-value	B (95% CI)	p-value	B (95% CI)	p-value	B (95% CI)	p-value	B (95% CI)	p-value	B (95% CI)	p-value
Female		0.62 (0.18 to 1.07)	**0.006**	0.19 (− 0.31 to 0.68)	0.45	0.79 (0.22 to 1.36)	**0.007**	0.57 (0.11 to 1.03)	**0.016**	0.20 (− 0.36 to 0.76)	0.48	1.27 (0.81 to 1.74)	** < 0.001**	0.93 (0.50 to 1.37)	** < 0.001**	1.12 (0.77 to 1.47)	**0.000**
Age		− 0.04 (− 0.06 to − 0.03)	** < 0.001**	− 0.03 (− 0.05 to − 0.01)	**0.001**	− 0.09 (− 0.11 to − 0.07)	** < 0.001**	− 0.06 (− 0.07 to − 0.04)	** < 0.001**	− 0.08 (− 0.10 to − 0.06)	** < 0.001**	− 0.09 (− 0.11 to − 0.08)	** < 0.001**	− 0.08 (− 0.10 to − 0.07)	** < 0.001**	− 0.12 (− 0.13 to − 0.10)	**0.000**
BMI		− 0.05 (− 0.1 to 0.01)	0.104	− 0.02 (− 0.08 to 0.04)	0.47	− 0.04 (− 0.11 to 0.02)	0.21	− 0.03 (− 0.09 to 0.02)	0.24	− 0.02 (− 0.09 to 0.04)	0.49	− 0.03 (− 0.09 to 0.02)	0.22	− 0.05 (− 0.11 to 0.00)	**0.044**	− 0.02 (− 0.06 to 0.02)	0.26
Vaccine type (base = Pfizer)		− 0.7 (− 1.17 to − 0.23)	**0.003**	0.57 (0.13 to 1.01)	**0.011**	− 0.75 (− 1.41 to − 0.09)	**0.025**	− 0.06 (− 0.5 to 0.38)	0.79	0.33 (− 0.19 to 0.84)	0.22	− 0.31 (− 0.78 to 0.15)	0.189	0.01 (− 0.41 to 0.42)	0.97	0.15 (− 0.18 to 0.48)	0.38
Smoking		− 0.35 (− 0.88 to 0.19)	0.20	− 0.39 (− 0.97 to 0.18)	0.181	− 0.73 (− 1.45 to 0.00)	**0.052**	− 0.03 (− 0.54 to 0.49)	0.92	− 0.58 (− 1.22 to 0.06)	0.078	− 0.69 (− 1.26 to − 0.13)	**0.033**	− 0.06 (− 0.54 to 0.42)	0.80	− 0.41 (− 0.80 to − 0.01)	**0.044**
Alcohol		0.24 (− 0.18 to 0.67)	0.26	0.44 (− 0.04 to 0.91)	0.072	0.18 (− 0.36 to 0.72)	0.52	0.13 (− 0.31 to 0.57)	0.56	0.09 (− 0.44 to 0.61)	0.74	0.46 (0.04 to 0.89)	**0.033**	0.22 (− 0.19 to 0.63)	0.28	0.20 (− 0.13 to 0.53)	0.24
Steroids		− 0.26 (− 1.6 to 1.07)	0.70	− 1.01 (− 3.12 to 1.10)	0.35	− 1.82 (− 4.48 to 0.84)	0.180	− 0.80 (− 2.27 to 0.68)	0.29	− 15.05 (− 2566.91 to 2536.81)	0.99	− 16.22 (− 1713.08 to 1680.64)	0.99	− 0.49 (− 1.88 to 0.90)	0.49	− 1.34 (− 2.70 to 0.01)	0.053
Biologics		− 14.28 (− 3545.89 to 3517.33)	0.99	0.80 (− 1.59 to 3.19)	0.51	1.47 (− 1.04 to 3.99)	0.25	0.17 (− 2.3 to 2.65)	0.89	− 13.94 (− 4233.31 to 4205.42)	1.00	1.12 (− 1.36 to 3.6)	0.26	0.91 (− − 1.09 to 2.90)	0.37	− 0.39 (− 2.86 to 2.09)	0.76
Hypertension		− 0.06 (− 0.54 to 0.41)	0.79	− 0.10 (− 0.61 to 0.41)	0.69	− 0.07 (− 0.81 to 0.68)	0.86	0.05 (− 0.46 to 0.56)	0.85	0.52 (− 0.12 to 1.17)	0.11	0.00 (− 0.57 to 0.57)	0.96	− 0.09 (− 0.60 to 0.43)	0.75	0.01 (− 0.40 to 0.42)	0.97
Immunosuppression		− 14.77 (− 2617.57 to 2588.02)	0.99	− 14.71 (− 3320.94 to 3291.52)	0.99	0.63 (− 2.16 to 3.42)	0.66	0.53 (− 1.34 to 2.39)	0.58	− 13.60 (− 3227.60 to 3200.39)	0.99	1.24 (− 1.12 to 3.60)	0.30	0.11 (− 1.9 to 2.11)	0.92	0.27 (− 1.57 to 2.12)	0.77
Anticancer drugs		0.6 (− 1.79 to 2.99)	0.63	− 18.29 (− 25,723.2 to 25,686.61)	1.00	2.36 (0.33 to 4.40)	**0.023**	0.57 (− 1.76 to 2.90)	0.63	− 17.87 (− 28,652.33 to 28,616.60)	1.00	1.23 (− 1.28 to 3.74)	0.34	− 18.36 (− 25,998.17 to 25,961.45)	1.00	0.34 (− 2.07 to 2.75)	0.78
Diabetes		− 0.07 (− 0.78 to 0.65)	0.85	− 0.17 (− 0.89 to 0.55)	0.64	− 0.05 (− 1.19 to 1.09)	0.94	0.37 (− 0.31 to 1.04)	0.29	0.48 (− 0.35 to 1.31)	0.26	− 0.63 (− 1.73 to 0.47)	0.26	0.35 (− 0.37 to 1.07)	0.40	− 0.32 (− 0.98 to 0.34)	0.34
Asthma		0.23 (− 0.96 to 1.43)	0.70	0.53 (− 0.66 to 1.72)	0.38	1.02 (− 0.23 to 2.27)	0.109	1.40 (0.47 to 2.33)	**0.003**	0.61 (− 0.74 to 1.96)	0.37	1.16 (0.15 to 2.17)	**0.024**	0.58 (− 0.50 to 1.66)	0.29	1 (0.15 to 1.86)	**0.021**
Dyslipidemia		0.29 (− 0.24 to 0.83)	0.29	0.45 (− 0.11 to 1.01)	0.118	− 0.16 (− 1.08 to 0.77)	0.74	− 0.27 (− 0.93 to 0.39)	0.43	− 0.05 (− 0.84 to 0.74)	0.90	0.02 (− 0.66 to 0.70)	0.95	0.08 (− 0.53 to 0.69)	0.79	0.41 (− 0.06 to 0.87)	0.086

Significant values are in bold.

### Association between systemic adverse reactions and IgG titer against the S protein

We used multiple regression analysis to predict variables that affected IgG antibodies, Nab, and ELISpot at the peak after the third vaccination (T1) (Table 3). Significantly higher IgG(S) at T1 was associated with males, BMI, third vaccination type (Moderna), Groups 5–9, and smoking. Furthermore, fold changes in IgG(S) between T1 and T2 were larger in the groups with fewer systemic adverse reactions (Group 1: 0.36, Group 2: 0.42, and Group 3: 0.41), smaller in groups with multiple systemic adverse reactions after the third vaccination (Group 6: 0.52, Group 8: 0.47, Group 9: 0.46, and Groups 6, 8, and 9: 0.47), and multiple systemic adverse reactions only after the second vaccination (Group 4: 0.47) (Table 4 and Supplementary Table S4). The geometric mean of IgG(S) at T2 was the smallest in the group without systemic adverse reactions after the third vaccination (Group 1: 630.7 AU/mL, Group 2: 734.2 AU/mL, Group 4: 772.4 AU/mL, and Group 1, 2, and 4: 668.2 AU/mL).

**Table 3:** Relationship between the groups and peak humoral or cellular immunity after the third vaccination.

	IgG(S) (N = 2195)		Nab (N = 2160)		T spot (N = 1047)	
	B (95% CI)	p-value	B (95% CI)	p-value	B (95% CI)	p-value
Female	− 308.05 (− 451.06 to − 165.04)	** < 0.001**	− 9.11 (− 22.33 to 4.12)	0.177	− 0.48 (− 2.92 to 1.95)	0.70
Age	− 4.64 (− 9.70 to 0.42)	0.072	− 0.85 (− 1.31 to − 0.38)	** < 0.001**	− 0.17 (− 0.26 to − 0.08)	** < 0.001**
BMI	38.94 (22.44 to 55.44)	** < 0.001**	1.92 (0.40 to 3.45)	**0.013**	− 0.07 (− 0.34 to 0.2)	0.60
Vaccine type (Moderna)	1054.17 (914.52 to 1193.81)	** < 0.001**	12.41 (− 0.51 to 25.33)	0.06	1.87 (− 0.45 to 4.18)	0.114
Group 1						
Group 2	140.95 (− 121.13 to 403.04)	0.29	21.89 (− 2.34 to 46.11)	0.077	2.52 (− 1.88 to 6.92)	0.26
Group 3	194.46 (− 94.93 to 483.86)	0.188	37.36 (10.61 to 64.11)	**0.006**	1.83 (− 3.04 to 6.71)	0.46
Group 4	34.56 (− 309.03 to 378.15)	0.84	2.17 (− 29.58 to 33.92)	0.89	5.24 (− 0.74 to 11.22)	0.086
Group 5	322.89 (47.89 to 597.88)	**0.021**	29.32 (3.90 to 54.74)	**0.024**	7.73 (3.26 to 12.21)	**0.001**
Group 6	833.86 (501.17 to 1166.54)	** < 0.001**	36.52 (5.77 to 67.27)	**0.020**	10.52 (4.40 to 16.64)	**0.001**
Group 7	374.92 (104.04 to 645.80)	**0.007**	35.54 (10.5 to 60.58)	**0.005**	8.00 (3.57 to 12.42)	** < 0.001**
Group 8	785.7 (529.73 to 1041.66)	** < 0.001**	38.39 (14.67 to 62.11)	**0.002**	15.19 (11.13 to 19.24)	** < 0.001**
Group 9	667.32 (461.01 to 873.62)	** < 0.001**	37.04 (17.96 to 56.12)	** < 0.001**	12.78 (9.31 to 16.25)	** < 0.001**
Interval between second and third vaccination	− 0.63 (− 4.09 to 2.82)	0.72	− 0.34 (− 0.66 to − 0.02)	**0.038**	0.04 (− 0.03 to 0.10)	0.26
Smoking	− 220.45 (− 387.36 to − 53.53)	**0.01**	− 9.17 (− 24.62 to 6.28)	0.25	− 3.38 (− 6.25 to − 0.51)	**0.021**
Alcohol	− 86.54 (− 220.38 to 47.29)	0.21	1.82 (− 10.56 to 14.20)	0.77	1.79 (− 0.48 to 4.06)	0.123
Steroids	− 77.61 (− 615.21 to 459.99)	0.78	− 82.92 (− 132.59 to − 33.25)	**0.001**	4.66 (− 3.16 to 12.48)	0.24
Biologicals	− 726.81 (− 1608.64 to 155.02)	0.106	− 139.8 (− 221.28 to − 58.32)	**0.001**	− 6.07 (− 20.72 to 8.59)	0.42
NSAIDs	− 17.76 (− 279.73 to 244.20)	0.89	− 41.96 (− 66.26 to − 17.67)	**0.001**	− 0.10 (− 4.38 to 4.18)	0.96
Immunosuppression	− 538.08 (− 1300.90 to 224.74)	0.167	− 79.86 (− 150.34 to − 9.38)	**0.026**	− 11.74 (− 23.17 to − 0.31)	**0.044**
Anticancer drugs	171.09 (− 730.55 to 1072.74)	0.71	− 73.35 (− 156.65 to 9.96)	0.084	− 4.26 (− 19.54 to 11.01)	0.58
Hypertension	− 103.81 (− 275.6 to 67.99)	0.24	1.31 (− 14.58 to 17.19)	0.87	0.59 (− 2.19 to 3.37)	0.68
Diabetes	− 195.99 (− 445.94 to 53.96)	0.124	− 9.23 (− 32.33 to 13.87)	0.43	− 1.48 (− 5.46 to 2.50)	0.47
Asthma	− 108.20 (− 416.28 to 199.89)	0.49	− 1.66 (− 30.12 to 26.81)	0.91	− 0.74 (− 6.10 to 4.62)	0.79
Dyslipidemia	147.64 (− 52.36 to 347.64)	0.148	9.35 (− 9.13 to 27.83)	0.32	0.76 (− 2.84 to 4.36)	0.68

Significant values are in bold.

**Table 4:** Reduction rates of the geometric mean (95% CI) of IgG antibodies, neutralizing antibodies, and T spot(S).

Group (number of systemic adverse reactions after the second and third vaccinations)	T1	T2	T2/T1
IgG(S) (N = 2041)			
Group 1 (0 and 0)	1739.0 (1623–1863.2)	630.7 (579.9–686.1)	0.36
Group 2 (1 and 0)	1743.3 (1549.2–1961.8)	734.2 (637.8–845.2)	0.42
Group 3 (0 and 1)	2079.9 (1806.5–2394.7)	856.3 (746.2–982.7)	0.41
Group 4 (multiple and 0)	1634.0 (1361.3–1961.2)	772.4 (652.0–915.1)	0.47
Group 5 (1 and 1)	2023.7 (1797.3–2278.6)	854.0 (737.0–989.6)	0.42
Group 6 (0 and multiple)	2595.8 (2214.3–3043.0)	1356.5 (1120.6–1641.9)	0.52
Group 7 (multiple and 1)	2045.7 (1850.4–2261.5)	884.0 (786.4–993.6)	0.43
Group 8 (1 and multiple)	2451.3 (2207.6–2721.9)	1145.4 (1020.7–1285.4)	0.47
Group 9 (multiple and multiple)	2436.1 (2305.7–2573.9)	1130.4 (1055.8–1210.2)	0.46
Nab (N = 2039)			
Group 1 (0 and 0)	672.0 (641.3–704.2)	413.0 (377.7–451.6)	0.61
Group 2 (1 and 0)	723.2 (690.9–757.1)	482.6 (430.1–541.5)	0.67
Group 3 (0 and 1)	733.1 (673.7–797.7)	555.7 (501.8–615.4)	0.76
Group 4 (multiple and 1)	676.6 (606.4–754.9)	488.8 (417.9–571.6)	0.72
Group 5 (1 and 1)	726.2 (684.1–771.0)	545.7 (486.7–612.0)	0.75
Group 6 (0 and multiple)	733.9 (668.4–805.8)	675.3 (616.1–740.2)	0.92
Group 7 (multiple and 1)	745.5 (705.2–788.2)	573.5 (524.0–627.8)	0.77
Group 8 (1 and multiple)	744.8 (699.8–792.6)	643.6 (597.6–693.3)	0.86
Group 9 (multiple and multiple)	757.5 (737.9–777.6)	619.4 (593.0–646.9)	0.82
T spot (N = 979)			
Group 1 (0 and 0)	6.4 (5.5–7.3)	5.5 (4.8–6.3)	0.86
Group 2 (1 and 0)	8.0 (6.1–10.4)	6.8 (5.3–8.8)	0.86
Group 3 (0 and 1)	9.1 (7.2–11.6)	7.6 (6.0–9.7)	0.84
Group 4 (multiple and 1)	11.5 (8.8–14.9)	10.5 (7.6–14.7)	0.92
Group 5 (1 and 1)	10.6 (8.1–14)	8.9 (6.8–11.5)	0.83
Group 6 (0 and multiple)	17.1 (12.4–23.6)	12.0 (8.6–16.8)	0.70
Group 7 (multiple and 1)	13.4 (10.8–16.6)	11.9 (9.5–14.8)	0.89
Group 8 (1 and multiple)	19.4 (16.2–23.1)	15.6 (12.7–19.2)	0.81
Group 9 (multiple and multiple)	17.9 (16.0–20.0)	14.3 (12.6–16.4)	0.80

### Associations between adverse reaction and Nab

Significantly, higher Nab at T1 was associated with BMI in Groups 3 and 5–9. Conversely, significantly lower Nab at T1 was associated with age, the interval between the second and third vaccinations, steroids, biologicals, NSAIDs, and immunosuppression. In addition, fold changes in Nab were larger in the group without systemic adverse reactions after the third vaccination (Group 1: 0.61, Group 2: 0.67, Group 4: 0.72, and Groups 1, 2, and 4: 0.64). These groups had the lowest geometric mean of Nab at T2 (Group 1: 413.0 AU/mL, Group 2: 482.6 AU/mL, Group 4: 488.8 AU/mL, and Groups 1, 2, and 4: 435.9 AU/mL). Fold changes in Nab were smaller in the groups with multiple systemic adverse reactions after the third vaccination (Group 6: 0.92, Group 8: 0.86, Group 9: 0.82, and Groups 6, 8, and 9: 0.84).

### Association between adverse reactions and cellar immunity

Peak ELISpot at T1 was significantly associated with age, Groups 5–9, and smoking habits. The geometric mean of ELISpot at T2 was the largest in the group with multiple systemic adverse reactions after the third vaccination (Group 6: 12.0, Group 8: 15.6, Group 9: 14.3, and Groups 6, 8, and 9: 14.4).

## Discussion

We investigated the pattern of systemic adverse reactions after the second and third COVID-19 vaccinations and their short- and long-term relationship with IgG(S), Nab, and ELISpot to clarify the impact of adverse reactions on immune dynamics.

The pattern of systemic adverse reactions was associated with higher peak values of humoral and cellular immunity after the third vaccination. Groups with systemic adverse reactions in both the second and third vaccinations (Groups 5, 7, 8, and 9) and multiple systemic adverse reactions only after the third vaccination (Group 6) were associated with significantly higher IgG(S), Nab, and ELISpot in the peak phase (T1). The group with one systemic adverse reaction only after the third vaccination (Group 3) was associated only with peak values of Nab. The results for Groups 5–9 were consistent with previous reports that systemic adverse reactions were associated with higher antibody titers^17–21^. However, the results of Group 3 were consistent with reports that denied any association between adverse reactions and humoral immunity^22–24^. Immune dynamics after the third vaccination may differ depending on the experience and number of systemic adverse reactions.

Those who experienced multiple systemic adverse reactions after the third vaccination achieved high levels of humoral and cellular immunity, which was maintained over 3 months. Overall, 930 (42.3%) participants who experienced multiple systemic adverse reactions after the third vaccination (Groups 6, 8, and 9) had significantly higher peak values of humoral and cellular immunity, and the fold change in humoral immune levels between T1 and T2 was small. Notably, Groups 6, 8, and 9 had the largest geometric mean of cellar immunity at T2. Previous studies have reported an association between adverse reactions and the long-term kinetics of humoral immunity^10^. Studies examining the relationship between adverse reactions and the long-term dynamics of cellular immunity were limited. However, systemic adverse reactions were predominantly related to achieving and maintaining humoral and cellular immunity. Therefore, this information may help promote uptake of a third vaccination, even for those who hesitate to receive the vaccination because of concerns about adverse reactions.

The group without systemic symptoms (Group 1), who were significantly older than all the groups, had the lowest peak values and largest reduction in humoral immunity and could not induce cellular immunity. Group 1 included 539 (24.5%) participants, who were significantly older than all the groups and significantly more likely to be male than Groups 2, 7, 8, and 9. Older age was significantly associated with lower peaks of Nab and ELISpot after the third vaccination. The result that older participants and men had fewer adverse reactions was consistent with previous reports^37–39^. Previous studies have also reported that older patients had smaller antigen-specific memory B cell and antigen-specific memory T cell responses, and reduction of humoral immunity^13,40–43^. People who are less likely to have adverse reactions, including the elderly, may have less antibody and cytokine production by antigen-specific memory B and T cells. Therefore, they may need to continue infection control measures and discuss further vaccination. In addition, identifying those who are more likely to experience systemic adverse reactions and investigating their long-term effects may be crucial in preventing excessive adverse reactions.

This study had some limitations that should be considered when interpreting the results. First, we used the Wuhan strain of pseudo virus to measure humoral and cellular immunity. Therefore, we could not assess whether efficacy against the mutant strains differed among the groups, making it difficult to consider a fourth and subsequent vaccination. Further research is needed to determine the impact of adverse reactions and immune dynamics on the variant strains of SARS-CoV-2. Second, adverse reactions were self-reported and could not be evaluated based on the severity of each symptom or objective measures. Third, cellular immunity did not significantly change in any of the groups between T1 and T2, making it difficult to discuss the fold change. Despite these limitations, this study was the first to investigate systemic adverse reactions and their short- and long-term relationships to humoral and cellular immunity. They may need to continue infection control measures and discuss further vaccination.

In conclusion, systemic adverse reactions after the third vaccination were beneficial in achieving high peak values and maintaining humoral and cellular immunity. This information may help promote uptake of a third vaccination, even for those who hesitate to receive the vaccine because of concerns about adverse reactions. In contrast, the population without systemic symptoms was associated with older age, males, and vulnerability in acquiring humoral and cellular immunity. They may need to continue infection control measures and discuss further vaccination.

## Methods

### Study participants

This was an observational historical cohort study. The study participants were recruited from healthcare workers, government office staff, residents, and nursing home residents in Ishikawa Country, Soma City, and Minamisoma City in Fukushima Prefecture. The recruitment of participants, blood sampling, and questionnaire surveys were conducted in cooperation with hospital groups and municipalities in the central and Soso areas of Fukushima Prefecture. This area has been continually testing for antibodies in healthcare workers and residents since 2020 to identify their infection status and control infections.

### Eligibility criteria

The inclusion criteria were as follows: (i) completed the third COVID-19 mRNA vaccination (BNT162b2 or mRNA-1273); (ii) completed blood sampling at T1 and T2. Overall, 2368 individuals met the criteria for (i) and (ii). We excluded (iii) 114 patients without a record of adverse reactions after the second or third vaccination and (iv) 56 patients who self-reported COVID-19 by June 2022 (T2). Finally, 2198 participants were included in the study. The ethics committees of Hirata Central Hospital (number 2021-0611-1) and Fukushima Medical University approved this study (number 2021-116). This study conformed to the Code of Ethics of the World Medical Association (Declaration of Helsinki). Written informed consent was obtained from all participants. This study was supported by the Japan Agency for Medical Research and Development and was conducted as part of the FVCS.

### Study design

**Figure 2:**
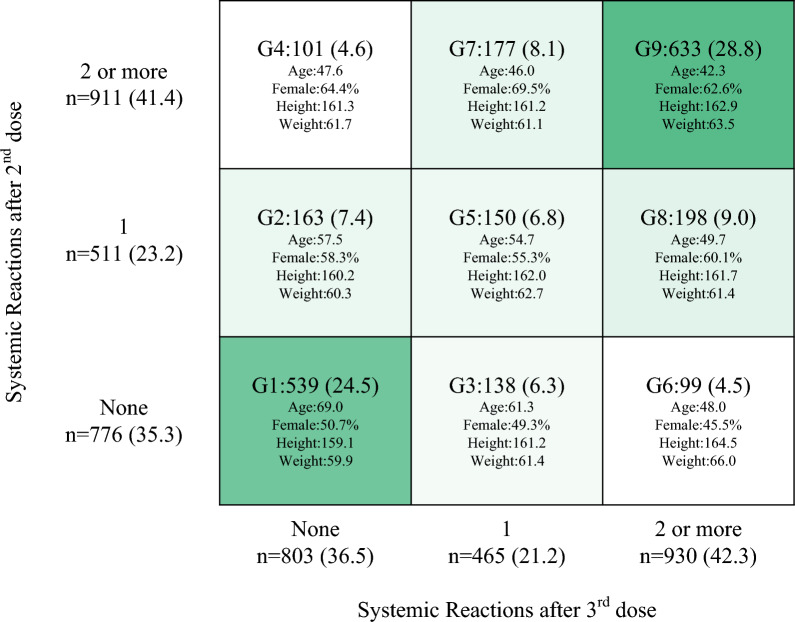
Groups according to the number of systemic adverse reactions after the second and third vaccinations (N = 2199). We classified participants into nine groups according to the number of systemic adverse reactions (0, 1, or more than 2) after the second and third vaccinations. Systemic adverse reactions were defined as fatigue, headache, muscle/joint pain, diarrhea, nausea, and dizziness. Group numbers were defined so that the larger the number of systemic adverse reactions, the larger the group number; for example, G1 was for those without systemic adverse reactions after the second and third vaccinations, and G9 was for those with multiple systemic adverse reactions after both vaccinations, among others.

We collected information from participants on age, sex, weight, height, alcohol consumption, smoking habits, medications, comorbidities, second and third vaccination types, and adverse reactions after the second and third vaccinations from a questionnaire survey. Regarding adverse reactions, the patients were asked about local pain, fever, headache, muscle/joint pain, diarrhea, nausea, and dizziness. Of the 2198 individuals who met the study eligibility criteria, at T1, 2195, 2160, and 1047 completed measurements for IgG(S), Nab, and ELISpot, respectively; at both T1 and T2, 2041, 2039, and 979 completed measurements for IgG(S), Nab, and ELISpot, respectively (Fig. 2).

### Cellular immune response

We evaluated cellular immune responses by ELISpot using T-spot COVID (Oxford Immunotec; UK). Blood samples were transferred from the hospital to LSI medicine within the blood sampling day; subsequently, all tests were performed in LSI medicine as per official guidelines. Effector T- cells generating interferon-gamma were counted as spots on the wells. The results were assessed by comparing the positive and negative control wells. The number of spots was counted up to 50; thus, more than 50 spots were shown as 50 and over. In addition, more than seven spots was evaluated as reactive, 5, 6, and 7 spots were evaluated as borderline, and fewer than five spots were evaluated as not reactive and complied following the official guidelines. The target antigen of ELISpot was the spike protein.

### Serological assay

Serological assays for IgG(S) and Nab were performed using the chemiluminescence immunoassay with iFlash 3000 (YHLO Biotech, Shenzhen, China) and iFlash-2019-nCoV series (YHLO Biotech, Shenzhen, China) as reagents at Tokyo University. A chemiluminescence immunoassay is used to quantitatively determine humoral immunity in human serum using an enzyme and chemiluminescence. The cutoff value of each assay (IgG against the S protein, Nab) was 10 AU/mL, following the manufacturer’s official cutoff values^44,45^. The cutoff values were determined using the receiver operating characteristic curve method. For Nab, values > 800 AU/mL were not guaranteed to be accurate, according to the manufacturer’s instructions. Testing was performed according to the official guidelines. Quality evaluations were conducted daily before starting measurements.

### Classification of participants

We classified the participants into nine groups according to the number of systemic adverse reactions (0, 1, or more than 2), after the second and third vaccinations, to investigate the effect of adverse reactions on immune dynamics (Table 5). Systemic adverse reactions were defined as fatigue, headache, muscle/joint pain, diarrhea, nausea, and dizziness. Group numbers were defined so that the larger the number of systemic adverse reactions, the larger the group number; for example, Group 1 was for those without systemic adverse reactions after the second and third vaccinations, and Group 9 was for those with two or more systemic adverse reactions in both vaccinations, among others.

**Table 5:** Patterns of systemic adverse reactions.

Group	Number of systemic adverse reactions	
	After the second vaccination	After the third vaccination
Group 1	0	0
Group 2	1	0
Group 3	0	1
Group 4	2 ≥	0
Group 5	1	1
Group 6	0	2 ≥
Group 7	2 ≥	1
Group 8	1	2 ≥
Group 9	2 ≥	2 ≥

### Statistical analysis

We compared the participants’ characteristics using descriptive statistics according to the nine patterns of systemic adverse reactions after the second and third vaccinations. Based on the BMI of the participants, < 18.5 was defined as thin, 18.5–25 as normal, and ≥ 25 as overweight. Categorical variables (sex, BMI, alcohol, smoking, daily medicine, comorbidity, vaccine type, and adverse reaction) were summarized as frequencies, and continuous variables (age, height, and weight) were summarized as means and standard deviations. First, a multinomial logistics regression analysis was performed to determine whether participants’ backgrounds differed among the nine groups based on the group without systemic adverse reactions (Group 1). Independent variables included sex, age, BMI, vaccine type, smoking habits, alcohol consumption, medications, and comorbidity in Groups 2–9. Second, a multiple regression analysis was performed to examine the relationship between the peak of immunity after the third vaccination and the pattern of systemic adverse reactions. The dependent variables were the values of IgG(S), Nab, and ELISpot at T1, and the independent variables included sex, age, BMI, type of third vaccination, Groups 1–9, the interval between the second and third vaccinations, smoking habits, alcohol consumption, medications, and comorbidities. Third, the geometric means of IgG(S), Nab, and ELISpot for each group were reported at T1 and T2. The fold change between the two-time points was calculated for the nine groups. Fourth, the participants’ ages were divided by 10 years, and the percentage of those who experienced one, or more than two, systemic adverse reactions after the second and third vaccinations was calculated for each weight and BMI. An IgG(S) antibody titer over 5000 arbitrary units per milliliter (AU/mL) was defined as 5000 AU/mL and Nab over 800 AU/mL was defined as 800 AU/mL for all analyses. Statistical significance was set at p < 0.05. All statistical analyses were performed using STATA/IC (version 15; Lightstone, DL, College Station, TX, USA) and Python (version 3.7.12).

## Supplementary Information

Supplementary Information.

## Data Availability

The data that supports the findings of this study are available from the corresponding author. However, restrictions apply to the availability of these data, which were used under license for the current study and are not publicly available. Data are, however, available upon reasonable request to the corresponding author and with permission from Fukushima Medical University School of Medicine.
