# Supplementary material for: Association of systemic adverse reaction patterns with long-term dynamics of humoral and cellular immunity after coronavirus disease 2019 third vaccination
Source: Sci Rep. 2023 Jun 7;13:9264. doi: 10.1038/s41598-023-36429-1 (PMC10246541; doi:10.1038/s41598-023-36429-1)
Supplement: Supplementary file 1 — Supplementary Information. [file 41598_2023_36429_MOESM1_ESM.docx]

Association of systemic adverse reaction patterns with long-term dynamics of humoral and cellular immunity after coronavirus disease 2019 third vaccination

Authors:

Makoto Yoshida ^1,2^, Yurie Kobashi ^2,3^, Takeshi Kawamura ^4,5^, Yuzo Shimazu ^2^, Yoshitaka Nishikawa ^3^, Fumiya Omata ^3^, Hiroaki Saito ^2^, Chika Yamamoto ^2^, Tianchen Zhao ^2^, Morihiro Takita ^2^, Naomi Ito ^2^, Kenji Tatsuno ^6^, Yudai Kaneko ^5,7^, Aya Nakayama ^4^, Tatsuhiko Kodama ^5^, Masatoshi Wakui ^8^, Kenzo Takahashi ^9,10^, Masaharu Tsubokura ^2,3,11*^

Supplementary Table S1. Adverse reactions after vaccination and baseline characteristics by group

|  | Group1 (n=539) | Group2 (n=163) | Group3 (n=138) | Group4 (n=101) | Group5 (n=150) | Group6 (n=99) | Group7 (n=177) | Group8 (n=198) | Group9 (n=633) |
| --- | --- | --- | --- | --- | --- | --- | --- | --- | --- |
| Age, mean [SD] | 69.0 [17.2] | 57.5 [16.4] | 61.3 [15.7] | 47.6 [15.2] | 54.7 [14.3] | 48 [17.3] | 46.0 [13.7] | 49.7 [13.9] | 42.3 [13.0] |
| Female | 273 (50.7) | 95 (58.3) | 68 (49.3) | 65 (64.4) | 83 (55.3) | 45 (45.5) | 123 (69.5) | 119 (60.1) | 396 (62.6) |
| Height, mean [SD] | 159.1 [11.1] | 160.2 [9.7] | 161.2 [8.9] | 161.3 [8.9] | 162.0 [10.2] | 164.5 [9.6] | 161.2 [8.7] | 161.7 [9.0] | 162.9 [8.9] |
| Weight, mean [SD] | 59.9 [14.4] | 60.3 [11.5] | 61.4 [12.4] | 61.7 [13.7] | 62.7 [13.3] | 66.0 [13.8] | 61.1 [14.0] | 61.4 [12.4] | 63.5 [14.0] |
| BMI |  |  |  |  |  |  |  |  |  |
| thin | 27 (5.0) | 9 (5.5) | 4 (2.9) | 8 (7.9) | 10 (6.7) | 2 (2.0) | 12 (7.6) | 11 (5.6) | 23 (3.6) |
| normal | 270 (50.1) | 99 (60.7) | 73 (52.9) | 48 (47.5) | 79 (52.7) | 52 (52.5) | 98 (62.0) | 113 (57.1) | 343 (54.2) |
| overweight | 145 (26.9) | 39 (23.9) | 38 (27.5) | 25 (24.8) | 43 (28.7) | 27 (27.3) | 48 (30.4) | 50 (25.3) | 171 (27.0) |
| Alcohol | 195 (36.2) | 74 (45.4) | 68 (49.3) | 44 (43.6) | 68 (45.3) | 49 (49.5) | 90 (50.9) | 88 (44.4) | 287 (45.3) |
| Smoking | 93 (17.3) | 27 (16.6) | 24 (17.4) | 14 (13.9) | 33 (22.0) | 17 (17.2) | 32 (18.1) | 46 (23.2) | 125 (19.7) |
| Daily medications |  |  |  |  |  |  |  |  |  |
| Steroids | 18 (3.4) | 3 (1.9) | 2 (1.5) | 2 (2.0) | 5 (3.4) | 1 (1.0) | 1 (0.6) | 4 (2.1) | 9 (1.4) |
| NSAIDs | 57 (10.8) | 12 (7.5) | 9 (6.7) | 9 (9.1) | 13 (8.9) | 11 (11.1) | 6 (3.4) | 10 (5.2) | 36 (5.8) |
| Acetaminophen | 20 (3.8) | 6 (3.8) | 1 (0.7) | 1 (1.0) | 2 (1.4) | 3 (3.0) | 2 (1.1) | 2 (1.0) | 15 (2.4) |
| Antihistamines | 26 (4.9) | 11 (6.8) | 7 (5.2) | 10 (9.9) | 9 (6.0) | 7 (7.1) | 8 (4.5) | 6 (3.1) | 51 (8.1) |
| Immunosuppression | 8 (1.5) | 0 (0.0) | 3 (2.3) | 1 (1.0) | 2 (1.4) | 0 (0.0) | 2 (1.1) | 2 (1.0) | 4 (0.6) |
| Biologics | 3 (0.6) | 0 (0.0) | 1 (0.8) | 1 (1.0) | 1 (0.7) | 0 (0.0) | 1 (0.6) | 2 (1.0) | 2 (0.3) |
| Anticancer drugs | 4 (0.8) | 1 (0.6) | 0 (0.0) | 2 (2.0) | 1 (0.7) | 0 (0.0) | 1 (0.6) | 0 (0.0) | 1 (0.2) |
| Comorbidities |  |  |  |  |  |  |  |  |  |
| Hypertension | 271 (50.4) | 52 (31.9) | 52 (37.7) | 15 (14.9) | 44 (29.3) | 26 (26.3) | 29 (16.4) | 40 (20.2) | 78 (12.3) |
| Diabetes | 71 (13.2) | 14 (8.6) | 14 (10.1) | 5 (5.0) | 14 (9.3) | 9 (9.1) | 5 (2.8) | 13 (6.6) | 20 (3.2) |
| Asthma | 17 (3.2) | 5 (3.1) | 4 (2.9) | 7 (6.9) | 12 (8.0) | 6 (6.1) | 10 (5.6) | 6 (3.0) | 39 (6.2) |
| Anaphylactic shock | 4 (0.7) | 1 (0.6) | 0 (0.0) | 1 (1.0) | 3 (2.0) | 0 (0.0) | 1 (0.6) | 0 (0.0) | 8 (1.3) |
| Gout | 23 (4.3) | 3 (1.8) | 4 (2.9) | 1 (1.0) | 4 (2.7) | 1 (1.0) | 5 (2.8) | 7 (3.5) | 16 (2.5) |
| Dyslipidemia | 81 (15.1) | 27 (16.6) | 26 (18.8) | 6 (5.9) | 14 (9.3) | 9 (9.1) | 14 (7.9) | 18 (9.1) | 56 (8.8) |
| Rheumatoid arthritis | 15 (2.8) | 4 (2.5) | 2 (1.4) | 2 (2.0) | 4 (2.7) | 0 (0.0) | 2 (1.1) | 3 (1.5) | 3 (0.5) |
| Respiratory disease | 17 (3.2) | 6 (3.7) | 3 (2.2) | 3 (3.0) | 1 (0.7) | 0 (0.0) | 2 (1.1) | 0 (0.0) | 13 (2.1) |
| Cardiovascular disease | 82 (15.2) | 18 (11.0) | 12 (8.7) | 3 (3.0) | 15 (10.0) | 3 (3.0) | 7 (4.0) | 7 (3.5) | 22 (3.5) |
| 2nd vaccination type (Moderna) | 0 (0.0) | 0 (0.0) | 0 (0.0) | 0 (0.0) | 0 (0.0) | 0 (0.0) | 0 (0.0) | 0 (0.0) | 1 (0.2) |
| Adverse reactions after 2nd vaccination |  |  |  |  |  |  |  |  |  |
| Local pain | 0 (0.0) | 80 (49.1) | 83 (60.1) | 53 (52.5) | 86 (57.3) | 73 (73.7) | 117 (66.1) | 119 (60.1) | 462 (73.0) |
| Fever | 0 (0.0) | 19 (11.7) | 0 (0.0) | 58 (57.4) | 14 (9.3) | 0 (0.0) | 98 (55.4) | 28 (14.1) | 393 (62.1) |
| Fatigue | 0 (0.0) | 77 (47.2) | 0 (0.0) | 86 (85.1) | 82 (54.7) | 0 (0.0) | 155 (87.6) | 115 (58.1) | 576 (91.0) |
| Headache | 0 (0.0) | 12 (7.4) | 0 (0.0) | 51 (50.5) | 9 (6.0) | 0 (0.0) | 91 (51.4) | 21 (10.6) | 399 (63.0) |
| Muscle/joint pain | 0 (0.0) | 49 (30.1) | 0 (0.0) | 59 (58.4) | 39 (26.0) | 0 (0.0) | 92 (52.0) | 29 (14.6) | 409 (64.6) |
| Diarrhea | 0 (0.0) | 1 (0.6) | 0 (0.0) | 4 (4.0) | 1 (0.7) | 0 (0.0) | 8 (4.5) | 2 (1.0) | 33 (5.2) |
| Nausea | 0 (0.0) | 2 (1.2) | 0 (0.0) | 8 (7.9) | 2 (1.3) | 0 (0.0) | 13 (7.3) | 1 (0.5) | 59 (9.3) |
| Dizziness | 0 (0.0) | 3 (1.8) | 0 (0.0) | 4 (4.0) | 3 (2.0) | 0 (0.0) | 10 (5.6) | 2 (1.0) | 72 (11.4) |
| 3rd vaccination type (Moderna) | 232 (43.2) | 35 (21.7) | 68 (49.6) | 19 (18.8) | 47 (31.5) | 39 (41.1) | 39 (22.0) | 60 (30.6) | 191 (30.5) |
| Adverse reactions after 3rd vaccination |  |  |  |  |  |  |  |  |  |
| Local pain | 235 (43.6) | 103 (63.2) | 71 (51.4) | 71 (70.3) | 83 (55.3) | 63 (63.6) | 121 (68.4) | 120 (60.6) | 491 (77.6) |
| Fever | 0 (0.0) | 0 (0.0) | 32 (23.2) | 0 (0.0) | 17 (11.3) | 63 (63.6) | 23 (13.0) | 108 (54.5) | 411 (64.9) |
| Fatigue | 0 (0.0) | 0 (0.0) | 51 (37.0) | 0 (0.0) | 71 (47.3) | 82 (82.8) | 93 (52.5) | 156 (78.8) | 568 (89.7) |
| Headache | 0 (0.0) | 0 (0.0) | 18 (13.0) | 0 (0.0) | 12 (8.0) | 53 (53.5) | 26 (14.7) | 106 (53.5) | 418 (66.0) |
| Muscle/joint pain | 0 (0.0) | 0 (0.0) | 35 (25.4) | 0 (0.0) | 47 (31.3) | 51 (51.5) | 33 (18.6) | 117 (59.1) | 415 (65.6) |
| Diarrhea | 0 (0.0) | 0 (0.0) | 1 (0.7) | 0 (0.0) | 1 (0.7) | 3 (3.0) | 2 (1.1) | 10 (5.1) | 37 (5.8) |
| Nausea | 0 (0.0) | 0 (0.0) | 0 (0.0) | 0 (0.0) | 0 (0.0) | 6 (6.1) | 0 (0.0) | 16 (8.1) | 70 (11.1) |
| Dizziness | 0 (0.0) | 0 (0.0) | 1 (0.7) | 0 (0.0) | 2 (1.3) | 8 (8.1) | 0 (0.0) | 11 (5.6) | 71 (11.2) |

NSAIDs, non-steroidal anti-inflammatory drugs; BMI, body mass index

Supplementary Table S2. Frequency of adverse reactions according to age and weight

|  |  |  | Systemic reactions after 2nd vaccination | | | Systemic reactions after 3rd vaccination | | |
| --- | --- | --- | --- | --- | --- | --- | --- | --- |
| **Age** | **Weight** | **n** | **1** | **2 or more** | **total** | **1** | **2 or more** | **total** |
| –19 | 50– | 5 | 0 (0.0) | 3 (60.0) | 3 (60.0) | 2 (40.0) | 1 (20.0) | 3 (60.0) |
|  | 60– | 1 | 0 (0.0) | 1 (100.0) | 1 (100.0) | 0 (0.0) | 1 (100.0) | 1 (100.0) |
|  | 70– | 1 | 0 (0.0) | 1 (100.0) | 1 (100.0) | 0 (0.0) | 1 (100.0) | 1 (100.0) |
|  | 80– | 3 | 0 (0.0) | 2 (66.7) | 2 (66.7) | 0 (0.0) | 2 (66.7) | 2 (66.7) |
| 20–29 | 30– | 2 | 0 (0.0) | 2 (100.0) | 2 (100.0) | 0 (0.0) | 2 (100.0) | 2 (100.0) |
|  | 40– | 27 | 7 (25.9) | 19 (70.4) | 26 (96.3) | 2 (7.4) | 23 (85.2) | 25 (92.6) |
|  | 50– | 40 | 4 (10.0) | 28 (70.0) | 32 (80.0) | 7 (17.5) | 29 (72.5) | 36 (90.0) |
|  | 60– | 43 | 8 (18.6) | 28 (65.1) | 36 (83.7) | 5 (11.6) | 30 (69.8) | 35 (81.4) |
|  | 70– | 24 | 5 (20.8) | 15 (62.5) | 20 (83.3) | 2 (8.3) | 16 (66.7) | 18 (75.0) |
|  | 80– | 23 | 7 (30.4) | 13 (56.5) | 20 (87.0) | 5 (21.7) | 15 (65.2) | 20 (86.9) |
| 30–39 | 40– | 28 | 3 (10.7) | 22 (78.6) | 25 (89.3) | 5 (17.9) | 18 (64.3) | 23 (82.2) |
|  | 50– | 78 | 17 (21.8) | 52 (66.7) | 69 (88.5) | 12 (15.4) | 51 (65.4) | 63 (80.8) |
|  | 60– | 87 | 18 (20.7) | 59 (67.8) | 77 (88.5) | 22 (25.3) | 51 (58.6) | 73 (83.9) |
|  | 70– | 49 | 9 (18.4) | 28 (57.1) | 37 (75.5) | 14 (28.6) | 26 (53.1) | 40 (81.7) |
|  | 80– | 49 | 8 (16.3) | 31 (63.3) | 39 (79.6) | 9 (18.4) | 30 (61.2) | 39 (79.6) |
| 40–49 | 40– | 40 | 6 (15.0) | 30 (75.0) | 36 (90.0) | 10 (25.0) | 17 (42.5) | 27 (67.5) |
|  | 50– | 114 | 32 (28.1) | 63 (55.3) | 95 (83.3) | 27 (23.7) | 60 (52.6) | 87 (76.3) |
|  | 60– | 102 | 32 (31.4) | 55 (53.9) | 87 (85.3) | 22 (21.6) | 58 (56.9) | 80 (78.5) |
|  | 70– | 70 | 14 (20.0) | 40 (57.1) | 54 (77.1) | 17 (24.3) | 36 (51.4) | 53 (75.7) |
|  | 80– | 59 | 17 (28.8) | 29 (49.2) | 46 (78.0) | 15 (25.4) | 34 (57.6) | 49 (83.0) |
| 50–59 | 30– | 3 | 1 (33.3) | 2 (66.7) | 3 (100.0) | 1 (33.3) | 1 (33.3) | 2 (66.6) |
|  | 40– | 34 | 18 (52.9) | 14 (41.2) | 32 (94.1) | 10 (29.4) | 15 (44.1) | 25 (73.5) |
|  | 50– | 119 | 38 (31.9) | 56 (47.1) | 94 (79.0) | 33 (27.7) | 59 (49.6) | 92 (77.3) |
|  | 60– | 84 | 23 (27.4) | 33 (39.3) | 56 (66.7) | 12 (14.3) | 45 (53.6) | 57 (67.9) |
|  | 70– | 60 | 13 (21.7) | 25 (41.7) | 38 (63.3) | 13 (21.7) | 30 (50.0) | 43 (71.7) |
|  | 80– | 35 | 8 (22.9) | 14 (40.0) | 22 (62.9) | 9 (25.7) | 13 (37.1) | 22 (62.8) |
| 60–69 | 30– | 3 | 1 (33.3) | 2 (66.7) | 3 (100.0) | 3 (100.0) | 0 (0.0) | 3 (100.0) |
|  | 40– | 44 | 13 (29.6) | 16 (36.4) | 29 (65.9) | 15 (34.1) | 14 (31.8) | 29 (65.9) |
|  | 50– | 112 | 36 (32.1) | 34 (30.4) | 70 (62.5) | 33 (29.5) | 39 (34.8) | 72 (64.3) |
|  | 60– | 107 | 34 (31.8) | 18 (16.8) | 52 (48.6) | 21 (19.6) | 25 (23.4) | 46 (43.0) |
|  | 70– | 43 | 7 (16.3) | 9 (20.9) | 16 (37.2) | 9 (20.9) | 9 (20.9) | 18 (41.8) |
|  | 80– | 24 | 4 (16.7) | 5 (20.8) | 9 (37.5) | 2 (8.3) | 7 (29.2) | 9 (37.5) |
| 70–79 | 30– | 3 | 0 (0.0) | 1 (33.3) | 1 (33.3) | 0 (0.0) | 1 (33.3) | 1 (33.3) |
|  | 40– | 34 | 11 (32.4) | 4 (11.8) | 15 (44.1) | 8 (23.5) | 6 (17.7) | 14 (41.2) |
|  | 50– | 70 | 21 (30.0) | 7 (10.0) | 28 (40.0) | 17 (24.3) | 10 (14.3) | 27 (38.6) |
|  | 60– | 68 | 13 (19.1) | 6 (8.8) | 19 (27.9) | 20 (29.4) | 8 (11.8) | 28 (41.2) |
|  | 70– | 39 | 7 (18.0) | 3 (7.7) | 10 (25.6) | 8 (20.5) | 4 (10.3) | 12 (30.8) |
|  | 80– | 6 | 2 (33.3) | 1 (16.7) | 3 (50.0) | 1 (16.7) | 2 (33.3) | 3 (50.0) |
| 80– | 20– | 2 | 0 (0.0) | 0 (0.0) | 0 (0.0) | 0 (0.0) | 0 (0.0) | 0 (0.0) |
|  | 30– | 26 | 0 (0.0) | 1 (3.9) | 1 (3.9) | 1 (3.9) | 1 (3.9) | 2 (7.8) |
|  | 40– | 82 | 8 (9.8) | 4 (4.9) | 12 (14.6) | 12 (14.6) | 4 (4.9) | 16 (19.5) |
|  | 50– | 49 | 9 (18.4) | 1 (2.0) | 10 (20.4) | 7 (14.3) | 2 (4.1) | 9 (18.4) |
|  | 60– | 12 | 3 (25.0) | 0 (0.0) | 3 (25.0) | 1 (8.3) | 0 (0.0) | 1 (8.3) |
|  | 70– | 6 | 0 (0.0) | 1 (16.7) | 1 (16.7) | 1 (16.7) | 0 (0.0) | 1 (16.7) |

Supplementary Table S3. Frequency of systemic adverse reactions by age and BMI

|  |  |  | Systemic reaction after 2nd dose | | | Systemic reaction after 3rd dose | | |
| --- | --- | --- | --- | --- | --- | --- | --- | --- |
| Age | BMI | n | 1 | 2 or more | Total | 1 | 2 or more | Total |
| –19 | Normal | 5 | 0 (0.0) | 3 (60.0) | 3 (60.0) | 1 (20.0) | 2 (40.0) | 3 (60.0) |
|  | Overweight | 5 | 0 (0.0) | 4 (80.0) | 4 (80.0) | 1 (20.0) | 3 (60.0) | 4 (80.0) |
| 20–29 | Thin | 15 | 3 (20.0) | 12 (80.0) | 15 (100.0) | 3 (20.0) | 10 (66.7) | 13 (86.7) |
|  | Normal | 96 | 16 (16.7) | 63 (65.6) | 79 (82.3) | 11 (11.5) | 73 (76.0) | 84 (87.5) |
|  | Overweight | 48 | 12 (25.0) | 30 (62.5) | 42 (87.5) | 7 (14.6) | 32 (66.7) | 39 (81.3) |
| 30–39 | Thin | 16 | 4 (25.0) | 11 (68.8) | 15 (93.8) | 4 (25.0) | 7 (43.8) | 11 (68.8) |
|  | Normal | 187 | 38 (20.3) | 124 (66.3) | 162 (86.6) | 36 (19.3) | 118 (63.1) | 154 (82.4) |
|  | Overweight | 87 | 13 (14.9) | 56 (64.4) | 69 (79.3) | 22 (25.3) | 50 (57.5) | 72 (82.8) |
| 40–49 | Thin | 13 | 6 (46.2) | 6 (46.2) | 12 (92.3) | 3 (23.1) | 8 (61.5) | 11 (84.6) |
|  | Normal | 243 | 59 (24.3) | 143 (59.7) | 202 (83.1) | 57 (23.5) | 128 (52.7) | 185 (76.1) |
|  | Overweight | 129 | 36 (27.9) | 68 (52.7) | 104 (80.6) | 31 (24.0) | 69 (53.5) | 100 (77.5) |
| 50–59 | Thin | 15 | 8 (53.3) | 6 (40.0) | 14 (93.3) | 3 (20.0) | 7 (46.7) | 10 (66.7) |
|  | Normal | 213 | 67 (31.5) | 88 (41.3) | 155 (72.8) | 51 (23.9) | 102 (47.9) | 153 (71.8) |
|  | Overweight | 106 | 26 (24.5) | 50 (47.2) | 76 (71.7) | 24 (22.6) | 54 (50.9) | 78 (73.6) |
| 60–69 | Thin | 16 | 4 (25.0) | 6 (37.5) | 10 (62.5) | 5 (31.3) | 4 (25.0) | 9 (56.3) |
|  | Normal | 208 | 63 (30.3) | 52 (25.0) | 115 (55.3) | 55 (26.4) | 59 (28.4) | 114 (54.8) |
|  | Overweight | 108 | 28 (25.9) | 26 (24.1) | 54 (50.0) | 22 (20.4) | 31 (28.7) | 53 (49.1) |
| 70–79 | Thin | 7 | 3 (42.9) | 1 (14.3) | 4 (57.2) | 4 (57.1) | 0 (0.0) | 4 (57.1) |
|  | Normal | 126 | 36 (28.6) | 12 (9.5) | 48 (38.1) | 30 (23.8) | 21 (16.7) | 51 (40.5) |
|  | Overweight | 78 | 13 (16.7) | 9 (11.5) | 22 (28.2) | 18 (23.1) | 9 (11.5) | 27 (34.6) |
| 80– | Thin | 24 | 2 (8.3) | 1 (4.2) | 3 (12.5) | 4 (16.7) | 0 (0.0) | 4 (16.7) |
|  | normal | 97 | 12 (12.4) | 4 (4.1) | 16 (16.5) | 9 (9.3) | 5 (5.2) | 14 (14.4) |
|  | overweight | 25 | 4 (16.0) | 1 (4.0) | 5 (20.0) | 4 (16.0) | 0 (0.0) | 4 (16.0) |

BMI, body mass index

Supplementary Table S4. Reduction rates of humoral and cellular immunity according to the number of systemic adverse reactions after the 3rd COVID-19 vaccination

| Number of systemic adverse reactions after the 3rd vaccination | T1 | T2 | T2/T1 |
| --- | --- | --- | --- |
| IgG(S) (N=2041) |  |  |  |
| 0 (Groups 1, 2, and 4) | 1726.0 (1630.8–1826.8) | 668.2 (625.1–714.2) | 0.39 |
| 1 (Groups 3,5, and 7) | 2048.8 (1914.4–2192.6) | 866.0 (802.4–934.6) | 0.42 |
| 2 ≥ (Groups 6, 8, and 9) | 2455.3 (2343.7–2572.3) | 1155.0 (1091.9–1221.7) | 0.47 |
| Nab (N=2039) |  |  |  |
| 0 (Groups 1, 2, and 4) | 682.9 (659.3–707.5) | 435.9 (407.6–466.1) | 0.64 |
| 1 (Groups 3,5, and 7) | 735.6 (708.3–764.0) | 559.2 (527.5–592.8) | 0.76 |
| 2 ≥ (Groups 6, 8, and 9) | 752.3 (734.3–770.7) | 630.1 (608.5–652.5) | 0.84 |
| Tspot (N=979) |  |  |  |
| 0 (Groups 1, 2, and 4) | 7.2 (6.4–8.0) | 6.3 (5.6–7.0) | 0.88 |
| 1 (Groups 3,5, and 7) | 11.2 (9.7–12.8) | 9.6 (8.3–11.0) | 0.86 |
| 2 ≥ (Groups 6, 8, and 9) | 18.2 (16.6–19.9) | 14.4 (13.0–16.0) | 0.79 |
